# Synthesis, Spectral Characterization, In-vitro Antibacterial and Antifungal Activities of Novel (2e)-Ethyl-2-(2-(2, 4-Dinitrophenyl) Hydrazono)-4-(Naphthalen-2-yl)-6-Arylcyclohex-3-Enecarboxylates

**Published:** 2011

**Authors:** V Kanagarajan, J Thanusu, M Gopalakrishnan

**Affiliations:** Synthetic Organic Chemistry Laboratory, Department of Chemistry, Annamalai University, Annamalai nagar-608 002, Tamil Nadu, India.

**Keywords:** Ethyl 4-(naphthalen-2-yl)-2-oxo-6-arylcyclohex-3-enecarboxylates, (2E)-ethyl-2-(2-(2, 4-dinitrophenyl)2hydrazono)-4-(naphthalen-2-yl) -6-arylcyclohex-3-enecarboxylates, 2, 4-Dinitrophenyl hydrazine, Antibacterial activity, Antifungal activity

## Abstract

In a search for new leads towards potent antimicrobial agents, an array of novel (2*E*)-ethyl-2-(2-(2,4-dinitrophenyl)hydrazono)-4-(naphthalen-2-yl)-6-arylcyclohex-3-ene carboxylates 17-24 were synthesized and characterized through their melting point, elemental analysis, MS, FT-IR, one-dimensional NMR (^1^H, D_2_O exchanged ^1^H and ^13^C), two dimensional HOMOCOR and HSQC spectroscopic data. *In-vitro *microbiological evaluations were carried out for all the newly synthesized compounds 17-24 against clinically isolated bacterial strains namely *Salmonella typhii, Klebsiellapneumoniae, Escherichia coli, Pseudomonas aeruginosa, Staphylococcus aureus, β-Hemolytic streptococcus *and *Micrococcus luteus*and also fungal strains namely *Aspergillusflavus, Aspergillusniger, Mucor, Rhizopus and Microsporumgypseum*and finally, the results of their structure activity relationship were discussed. The obtained results can be used as the key step for the building of novel chemical compounds with interesting antimicrobial profiles comparable to that of the standard drugs.

## Introduction

**Figure 1:**
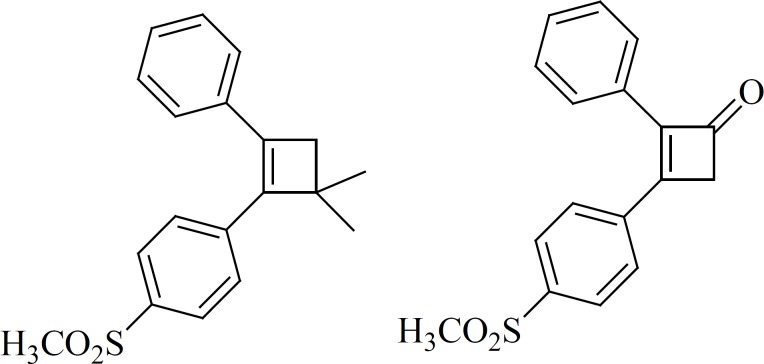
Structurally diverse clinically potent hydrazones that possess varied biological activities

Over the past few decades, health-related quality of human life benefits are under threat as many commonly used antibiotics have become less and less effective against certain illnesses, not only because many of them produce toxic reactions but also due to emergence of drug resistant microbes. Hydrazones constitute an important class of compounds for new drug development. Therefore, many researchers have synthesized these compounds as target structures and evaluated their biological activities. These observations have been conducted for the development of new hydrazones that possess varied biological activities (1). Some critical reviews have been published which give an outlook on the developments on antimycobacterialhydrazones (2-5). Hydrazones have been demonstrated to possess varied biological activities (Figure1). For example, isonicotinoylhydrazones (A) are antitubercular and nifuroxazide (B) is an intestinal antiseptic. Acetylhydrazones (C) provided a good protection against convulsions (6). The 3-Phenyl-5-sulfonamidoindole-2-carboxylic acid 3, 4-methylenedioxy/4-methyl/4-nitrobenzylidene-hydrazide (D) showed antidepressant activity (7). The most important anti-inflammatory derivative 2-(2-formylfuryl) pyridylhydrazone (E) presented a 79% inhibition of pleurisy (8). [(4′-N, N-Dimethylaminobenzylidene-3-(3′, 4′-ethylenedioxyphenyl) propionylhydrazine] (F) are used as standard antinociceptive drugs (9). The antiplatelet activity of novel tricyclic acylhydrazone derivatives (G) is evaluated by their ability to inhibit platelet aggregation of rabbit platelet-rich plasma (10). The aroylhydrazonechelator 2-hydroxy-1-naphthylaldehyde isonicotinoylhydrazone (H) showed a greater antimalarial agent activity than desferrioxamine against chloroquine-resistant and sensitive parasites (11). Triazolohydrazones (I) possess a good antibacterial activity (12). A new bioactive compound of N-acylhydrazone class, 3, 4-methylenedioxybenzoyl-2-thienyl hydrazone (J) named LASSBio-294, was shown to have inotropic and vasodilatory effects (13). Triarylhydrazones (K) exhibit antitumoral activity against MCF-7 and ZR-75-1 human malignant breast cell lines (14).

The 2-Acetyl naphthalene is an interesting starting material which is widely used in perfume formulations (15), mainly in Neroli orange blossom, sweet pea, magnolia, honeysuckle, wisteria, narcisse, jasmine, various exotic florals, *etc. *In flavor composition, also, the ketone finds a place in imitation of strawberry, grape, various citrus and berry compositions in neroli and other natural flavors, in fruit complexes and in certain types of vanilla flavor. Nowadays, there has been a great deal of interest in exploiting more than one proximal functional groups for designing novel structures capable of performing a variety of functions. Synthesis of molecules, which are novel yet resembling known biologically active molecules by virtue of the presence of some critical structural features, is an essential component of the search for new leads in drug designing programme. Taking these considerations into account and as a part of our research program aimed at the synthesis of bioactive novel structurally diverse heterocycles (16-20), herein is reported the molecular conjugation of the naphthyl substituted chalcones moiety with two or more active counterparts which has been designed and synthesized with the hope of producing novel ethyl 4-(naphthalen-2-yl)-2-oxo-6-arylcyclohex-3-enecarboxylates (10-17), an intermediate with three versatile functional groups *i.e., *ketone, olefin and ester for the synthesis of (2E)-ethyl-2-(2-(2, 4-dinitrophenyl)hydrazono)-4-(naphthalen-2-yl)-6-arylcyclohex-3-ene carboxylates 17-24, a novel hydrazone derivative and to study their *in-vitro *microbiological evaluation against clinically isolated bacterial and fungal strains.

## Experimental

*Chemistry*

The progress of the reaction is monitored by TLC analysis. All the reported melting points are taken in open capillaries and are uncorrected. IR spectra are recorded in KBr (pellet forms) on a Nicolet-Avatar–330 FT-IR spectrophotometer and note worthy absorption values (cm^-1^) alone are listed. ^1^H and ^13^C NMR spectra are recorded at 400 MHz and 100 MHz respectively on BrukerAvance II 400 NMR spectrometer using DMSO-*d*_6_ as solvent. Two dimensional HOMOCOR and HSQC spectra are recorded at Bruker DRX 500 NMR spectrometer. The ESI +ve MS spectra are recorded on a BrukerDaltonics LC-MS spectrometer. Satisfactory microanalyses are obtained on Carlo Erba 1106 CHN analyzer.

By adopting the literature precedent (21), (E)-1-naphthalen-2-yl)-3-arylprop-2-en-1-ones 1-8 were prepared.

*General procedure for the synthesis of ethyl 4-(naphthalen-2-yl)-2-oxo-6-arylcyclohex-3-enecarboxylates*, *compounds 9-16*

To a solution of sodium ethoxide (0.001 mol) in 30 mL of absolute ethanol, freshly distilled ethyl acetoacetate (0.01 mol) and respective (E)-1-naphthalen-2-yl)-3-arylprop-2-en-1-ones (0.01 mol) in absolute ethanol (40 mL) was mixed. This mixture was refluxed in a water bath for 1-3 h by maintaining the temperature around (70-80)ºC. The reaction mixture was allowed to be cooled and filtered. Then the crude product was recrystallized from absolute ethanol to afford ethyl 4-(naphthalen-2-yl)-2-oxo-6-arylcyclohex-3-enecarboxylates.

*Ethyl 4-(naphthalen-2-yl)-2-oxo-6-phenylcyclohex-3-enecarboxylate, compound 9*

IR (KBr) ν (cm^-1^): 3057, 2987, 2929, 1738, 1659, 1606, 1292, 1155, 847, 816, 766, 704; ^1^H NMR (δ ppm), (J Hz): 0.92 (3H, t, CH_2_C*H*_3_ at C-1, J = 7.1), 3.22-3.28 (2H, H_5_, m), 3.65-3.73 (1H, H_6_, m), 3.91 (2H, q, C*H*_2_CH_3_ at C-1, J=7.0), 4.17 (1H, H_1_, d, J = 13.4), 6.73 (1H, s, H_3_), 7.24-8.33 (12H, m, H_arom_.); ^13^C NMR (δ ppm): 13.7 CH_2_*C*H_3_ at C-1, 35.1 C-5, 43.8 C-6, 58.7 *C*H_2_CH_3_ at C-1, 59.9 C-1, 123.1 C-3, 169.2 C=O at C-1, 194.2 C-2, 123.4-128.8 -C_arom_., C-4 carbon may be merged with -C_arom_, 132.7, 133.6, 134.3, 141.5 *ipso*-C’s.

*Ethyl 4-(naphthalen-2-yl)-2-oxo-6-p-tolylcyclohex-3-enecarboxylate, compound 10*

IR (KBr) ν (cm^-1^): 3065, 3027, 2979, 2923, 2863, 1740, 1660, 1604, 1148, 897, 849, 816, 753; ^1^H NMR (δ ppm), (J Hz): 0.96 (3H, t, CH_2_C*H*_3_ at C-1, J = 7.1), 2.28 (3H, s, CH_3_ at phenyl ring), 3.15-3.17 (2H, H_5_, m), 3.62-3.66 (1H, H_6_, m), 3.92 (2H, q, C*H*_2_CH_3_ at C-1, J = 6.9), 4.11 (1H, H_1_, d, J = 13.3), 6.71 (1H, s, H_3_), 7.13-8.30 (11H, m, H_arom_.); ^13^C NMR (δ ppm): 14.2 CH_2_*C*H_3_ at C-1, 21.0 CH_3_ at phenyl ring, 35.7 C-5, 43.8 C-6, 59.2 *C*H_2_CH_3_ at C-1, 60.3 C-1, 123.5 C-3, 169.6 C=O at C-1, 194.7 C-2, 123.7-129.2 -C_arom_., C-4 carbon may be merged with -C_arom_, 133.1, 134.0, 134.7, 136.5, 138.9 *ipso*-C’s.

*Ethyl 6-(4-fluorophenyl)-4-(naphthalen-2-yl)-2-oxocyclohex-3-enecarboxylate, compound 11*

IR (KBr) ν (cm^-1^): 3054, 2989, 2925, 1739, 1656, 1603, 1225, 1153, 888, 841, 815, 752; ^1^H NMR (δ ppm), (J Hz): 0.94 (3H, t, CH_2_C*H*_3_ at C-1, J = 7.0), 3.19-3.21 (2H, H_5_, m), 3.68-3.75 (1H, H_6_, m), 3.93 (2H, q, C*H*_2_CH_3_ at C-1, J = 7.0), 4.16 (1H, H_1_, d, J = 13.4), 6.73 (1H, s, H3), 7.16-8.33 (11H, m, H_arom_.); ^13^C NMR (δ ppm): 13.8 CH_2_*C*H_3_ at C-1, 35.0 C-5, 43.0 C-6, 58.8 *C*H_2_CH_3_ at C-1, 59.9 C-1, 123.0 C-3, 169.1 C=O at C-1, 194.1 C-2, 114.9-129.6 -C_arom_., C-4 carbon may be merged with -C_arom_, 132.7, 1333.6, 134.2, 137.7, 160.0 *ipso*-C’s.

*Ethyl6-(4-methoxyphenyl)-4-(naphthalen-2-yl)-2-oxocyclohex-3-enecarboxylate, compound12*

IR (KBr) ν (cm^-1^): 3056, 2987, 2932, 2838, 1738, 1657, 1606, 1252, 888, 831, 815, 749; ^1^H NMR (δ ppm), (J Hz): 0.96 (3H, t, CH_2_C*H*_3_ at C-1, J = 7.0), 3.16-3.29 (2H, H_5_, m), 3.59-3.67 (1H, H_6_, m), 3.73 (3H, s, OCH_3_ at phenyl ring), 3.92 (2H, q, C*H*_2_CH_3_ at C-1, J = 7.0), 4.10 (1H, H_1_, d, J = 14.3), 6.71 (1H, s, H_3_), 6.89-8.32 (11H, m, H_arom_.); ^13^C NMR (δ ppm): 14.2 CH_2_*C*H_3_ at C-1, 35.7 C-5, 43.5 C-6, 55.4 OCH_3_ at phenyl ring, 59.5 *C*H_2_CH_3_ at C-1, 60.2 C-1, 123.5 C-3, 169.7 C=O at C-1, 194.7 C-2, 114.1-129.0 -C_arom_., C-4 carbon may be merged with -C_arom_, 129.2, 133.1, 134.0, 134.7, 159.1 *ipso*-C’s.

*Ethyl 6-(4-chlorophenyl)-4-(naphthalen-2-yl)-2-oxocyclohex-3-enecarboxylate, compound 13*

IR (KBr) ν (cm^-1^): 3056, 3022, 2978, 2928, 1738, 1658, 1257, 1147, 890, 850, 818, 749; ^1^H NMR (δ ppm), (J Hz): 0.95 (3H, t, CH_2_C*H*_3_ at C-1, J = 7.0), 3.19-3.29 (2H, H_5_, m), 3.68-3.75 (1H, H_6_, m), 3.93 (2H, q, C*H*_2_CH_3_ at C-1, J=7.1), 4.18 (1H, H_1_, d, J = 13.4), 6.73 (1H, s, H_3_), 7.40-8.32 (11H, m, H_arom_.); ^13^C NMR (δ ppm): 13.8 CH_2_*C*H_3 _at C-1, 34.8 C-5, 43.1 C-6, 58.5 *C*H_2_CH_3_ at C-1, 60.0 C-1, 123.0 C-3, 169.1 C=O at C-1, 193.9 C-2, 123.3-129.5 -C_arom_., C-4 carbon may be merged with -C_arom_, 132.7, 133.6, 134.2, 134.9, 140.5 *ipso*-C’s.

*Ethyl 6-(4-bromophenyl)-4-(naphthalen-2-yl)-2-oxocyclohex-3-enecarboxylate*, *compound 14*

IR (KBr) ν (cm^-1^): 3058, 3027, 2977, 2929, 2907, 2863, 1732, 1657, 1598, 1170, 1145, 892, 817, 748, 711; ^1^H NMR (δ ppm), (J Hz): 0.96 (3H, t, CH_2_C*H*_3_ at C-1, J = 7.0), 3.19-3.21 (2H, H_5_, m), 3.67-3.74 (1H, H_6_, m), 3.94 (2H, q, C*H*_2_CH_3_ at C-1, J = 7.0), 4.18 (1H, H_1_, d, J = 13.4), 6.73 (1H, s, H_3_), 7.42-8.32 (11H, m, H_arom_.); ^13^C NMR (δ ppm): 13.8 CH_2_*C*H_3_ at C-1, 34.8 C-5, 43.2 C-6, 58.5 *C*H_2_CH_3_ at C-1, 60.0 C-1, 123.1 C-3, 169.1 C=O at C-1, 194.0 C-2, 120.1-129.9 -C_arom_., C-4 carbon may be merged with -C_arom_, 131.3, 132.7, 133.6, 134.2, 141.0 *ipso*-C’s.

*Ethyl 4-(naphthalen-2-yl)-6-(4-nitrophenyl)-2-oxo-cyclohex-3-enecarboxylate, compound 15*

IR (KBr) ν (cm^-1^): 3057, 2978, 2925, 2852, 1734, 1658, 1599, 1278, 1149, 893, 856, 815, 743; ^1^H NMR (δ ppm), (J Hz): 0.94 (3H, t, CH_2_C*H*_3 _at C-1, J = 6.9), 3.17-3.26 (2H, H_5_, m), 3.68-3.73 (1H, H_6_, m), 3.92 (2H, q, C*H*_2_CH_3_ at C-1, J = 6.8), 4.17 (1H, H_1_, d, J = 13.7), 6.73 (1H, s, H_3_), 6.98-8.22 (11H, m, H_arom_.); ^13^C NMR (δ ppm): 13.5 CH_2_*C*H_3 _at C-1, 34.8 C-5, 43.3 C-6, 58.7 *C*H_2_CH_3_ at C-1, 60.3 C-1, 123.2 C-3, 169.8 C=O at C-1, 193.2 C-2, 123.8-129.1 -C_arom_., C-4 carbon may be merged with -C_arom_, 132.6, 133.6, 134.3, 134.8, 140.1 *ipso*-C’s.

*Ethyl 6-(3-chlorophenyl)-4-(naphthalen-2-yl)-2-oxocyclohex-3-enecarboxylate, compound 16*

IR (KBr) ν (cm^-1^): 3058, 2977, 2927, 2869, 1658, 1733, 1597, 1170, 1147, 888, 851, 815, 786; ^1^H NMR (δ ppm), (J Hz): 0.94 (3H, t, CH_2_C*H*_3_ at C-1, J = 7.1), 3.19-3.28 (2H, H_5_, m), 3.66-3.73 (1H, H_6_, m), 3.91 (2H, q, C*H*_2_CH_3_ at C-1, J = 7.1), 4.16 (1H, H_1_, d, J = 13.7), 6.74 (1H, s, H_3_), 7.08-8.29 (11H, m, H_arom_.); ^13^C NMR (δ ppm): 13.7 CH_2_*C*H_3_ at C-1, 34.8 C-5, 43.2 C-6, 58.6 *C*H_2_CH_3 _at C-1, 60.1 C-1, 123.2 C-3, 169.0 C=O at C-1, 193.7 C-2, 123.1-129.8 -C_arom_., C-4 carbon may be merged with -C_arom_, 132.5, 133.6, 134.0, 134.7, 140.3 *ipso*-C’s.

*General method for the synthesis of (2E)-ethyl-2-(2-(2,4-dinitrophenyl)hydrazono)-4-(naphthalen-2-yl)-6-arylcyclohex-3-enecarboxylates, compounds 17-24*

A solution of ethyl 4-(naphthalen-2-yl)-2-oxo-6-arylcyclohex-3-enecarboxylates 9-16 (0.001 mol) in ethanol (40 mL) was treated with 2,4-dintrophenylhydrazine (0.001 mol) and 2-4 drops of sulfuric acid and refluxed for 2-3 h. The reaction mixture was cooled and then poured over crushed ice. The crude products were recrystallized twice using ethanol as solvent to afford the product.

*(2E)-ethyl-2-(2-(2,4-dinitrophenylhydrazono)-4-(naphthalen-2-yl)-6-phenylcyclohex-3-enecarboxylate*, *compound 17*

IR (KBr) ν (cm^-1^): 3323, 3099, 3060, 2989, 2924, 2847, 1737, 1656, 1512, 1330, 818, 763, 704, 623; ^1^H NMR (δ ppm), (J Hz): 0.92 (3H, t, CH_2_C*H*_3_ at C-1, J = 7.0), 3.20-3.21 (2H, H_5_, m), 3.66-3.72 (1H, H_6_, m), 3.91 (2H, q, C*H*_2_CH_3_ at C-1, J = 7.0), 4.16 (1H, H_1_, d, J = 13.4), 6.73 (1H, s, H_3_), 7.25-8.80 (15H, m, H_arom_.), 9.97 (1H, bs, NH); In the D_2_O exchanged ^1^H NMR spectrum, a broad singlet at 9.97 ppm due to labile NH proton disappeared; ^13^C NMR (δ ppm): 13.8 CH_2_*C*H_3_ at C-1, 35.1 C-5, 43.8 C-6, 59.9 *C*H_2_CH_3_ at C-1, 58.7 C-1, 115.5 C-3, 169.2 C=O at C-1, 158.7 C-2, 123.1-128.8 -C_arom_., C-4 carbon may be merged with -C_arom_, 129.5, 132.7, 133.6, 134.3, 141.5, 149.5 *ipso*-C’s.

*(2E)-ethyl-2-(2-(2,4-dinitrophenylhydrazono)-4-(naphthalen-2-yl)-6-p-tolylcyclohex-3-enecarboxylate, compound 18*

IR (KBr) ν (cm^-1^): 3323, 3096, 3049, 3022, 2972, 2923, 2858, 1739, 1657, 1512, 1330, 817, 747, 705, 629; ^1^H NMR (δ ppm), (J Hz): 0.95 (3H, t, CH_2_C*H*_3_ at C-1, J = 7.0), 2.28 (3H, s, CH_3_ at phenyl ring), 3.15-3.17 (2H, H_5_, m), 3.60-3.68 (1H, H_6_, m), 3.92 (2H, q, C*H*_2_CH_3_ at C-1, J = 7.0), 4.12 (1H, H_1_, d, J = 13.4), 6.71 (1H, s, H_3_), 7.14-8.80 (14H, m, H_arom_.), 9.97 (1H, bs, NH); In the D_2_O exchanged ^1^H NMR spectrum, a broad singlet at 9.97 ppm due to labile NH proton disappeared; ^13^C NMR (δ ppm): 13.8 CH_2_*C*H_3_ at C-1, 20.6 CH_3_ at phenyl ring, 35.3 C-5, 43.4 C-6, 59.9 *C*H_2_CH_3_ at C-1, 58.9 C-1, 115.5 C-3, 169.3 C=O at C-1, 158.7 C-2, 123.1-128.9 -C_arom_., C-4 carbon may be merged with -C_arom_, 129.5, 132.7, 133.6, 134.3, 136.1, 138.6, 149.1 *ipso*-C’s.

*(2E)-ethyl-2-(2-(2,4-dinitrophenylhydrazono)-6-(4-fluorophenyl)-4-(naphthalen-2-yl)-cyclohex-3-enecarboxylate, compound 19*

IR (KBr) ν (cm^-1^): 3319, 3100, 3071, 2923, 2852, 1739, 1654, 1510, 1332, 839, 749, 704, 627; ^1^H NMR (δ ppm), (J Hz): 0.93 (3H, t, CH_2_C*H*_3_ at C-1, J = 7.0), 3.18-3.20 (2H, H_5_, m), 3.46-3.74 (1H, H_6_, m), 3.92 (2H, q, C*H*_2_CH_3_ at C-1, J = 7.0), 4.15 (1H, H_1_, d, J = 14.4), 6.72 (1H, s, H_3_), 7.15-8.80 (14H, m, H_arom_.), 9.96 (1H, bs, NH); In the D_2_O exchanged ^1^H NMR spectrum, a broad singlet at 9.96 ppm due to labile NH proton disappeared; ^13^C NMR (δ ppm): 13.8 CH_2_*C*H_3_ at C-1, 35.1 C-5, 43.1 C-6, 60.1 *C*H_2_CH_3_ at C-1, 59.0 C-1, 115.2 C-3, 169.5 C=O at C-1, 158.8 C-2, 115.0-129.6 -C_arom_., C-4 carbon may be merged with -C_arom_, 129.7, 132.7, 133.7, 134.3, 137.8, 149.2, 162.5 *ipso*-C’s.

*(2E)-ethyl-2-(2-(2,4-dinitrophenylhydrazono)-6-(4-methoxyphenyl)-4-(naphthalen-2-yl)-cyclohex-3-enecarboxylate, Compound 20*

IR (KBr) ν (cm^-1^): 3319, 3054, 2994, 2928, 2841, 1737, 1655, 1512, 1332, 828, 747, 704, 628; ^1^H NMR (δ ppm), (J Hz): 0.95 (3H, t, CH_2_C*H*_3_ at C-1, J = 7.1), 3.14-3.16 (2H, H_5_, m), 3.54-3.62 (1H, H_6_, m), 3.73 (3H, s, OCH_3_ at phenyl ring), 3.92 (2H, q, C*H*_2_CH_3_ at C-1, J=7.0), 4.08 (1H, H_1_, d, J = 13.4), 6.88 (1H, s, H_3_), 6.89-8.89 (14H, m, H_arom_.), 9.97 (1H, bs, NH); In the D_2_O exchanged ^1^H NMR spectrum, a broad singlet at 9.97 ppm due to labile NH proton disappeared; ^13^C NMR (δ ppm): 13.8 CH_2_*C*H_3_ at C-1, 35.3 C-5, 43.1 C-6, 55.0 OCH_3_ at phenyl ring, 59.9 *C*H_2_CH_3_ at C-1, 59.2 C-1, 115.4 C-3, 169.4 C=O at C-1, 158.7 C-2, 113.7-129.4 -C_arom_., C-4 carbon may be merged with -C_arom_, 132.7, 133.0, 133.5, 133.6, 134.38, 134.30, 149.1, 158.3 *ipso*-C’s.

*(2E)-ethyl-2-(2-(2,4-dinitrophenylhydrazono)-6-(4-chlorophenyl)-4-(naphthalen-2-yl)-cyclohex-3-enecarboxylate, Compound 21*

IR (KBr) ν (cm^-1^): 3323, 3088, 2972, 2924, 2847, 1736, 1645, 1514, 1326, 823, 744, 629; ^1^H NMR (δ ppm), (J Hz): 0.92 (3H, t, CH_2_C*H*_3_ at C-1, J = 7.1), 3.15-3.20 (2H, H_5_, m), 3.69-3.75 (1H, H_6_, m), 3.95 (2H, q, C*H*_2_CH_3_ at C-1, J = 7.1), 4.16 (1H, H_1_, d, J = 13.4), 6.74 (1H, s, H_3_), 7.27-8.90 (14H, m, H_arom_.), 9.99 (1H, bs, NH); In the D_2_O exchanged ^1^H NMR spectrum, a broad singlet at 9.99 ppm due to labile NH proton disappeared; ^13^C NMR (δ ppm): 13.8 CH_2_*C*H_3_ at C-1, 34.9 C-5, 43.1 C-6, 60.0 *C*H_2_CH_3_ at C-1, 58.6 C-1, 115.4 C-3, 169.1 C=O at C-1, 158.6 C-2, 120.9-129.4 -C_arom_., C-4 carbon may be merged with -C_arom_, 129.5, 131.6, 132.7, 133.0, 133.6, 134.2, 140.5, 149.1 *ipso*-C’s.

*(2E)-ethyl-2-(2-(2,4-dinitrophenylhydrazono)-6-(4-bromophenyl)-4-(naphthalen-2-yl)-cyclohex-3-enecarboxylate, compound 22*

IR (KBr) ν (cm^-1^): 3324, 3097, 2978, 2923, 2847, 1735, 1643, 1514, 1330, 826, 744, 705, 629; ^1^H NMR (δ ppm), (J Hz): 0.95 (3H, t, CH_2_C*H*_3_ at C-1, J = 7.0), 3.15-3.20 (2H, H_5_, m), 3.57-3.73 (1H, H_6_, m), 3.93 (2H, q, C*H*_2_CH_3_ at C-1, J = 7.1), 4.17 (1H, H_1_, d, J = 14.4), 6.73 (1H, s, H_3_), 7.42-8.80 (14H, m, H_arom_.), 9.97 (1H, bs, NH); In the D_2_O exchanged ^1^H NMR spectrum, a broad singlet at 9.97 ppm due to labile NH proton disappeared; ^13^C NMR (δ ppm): 13.8 CH_2_*C*H_3 _at C-1, 34.8 C-5, 43.2 C-6, 60.1 *C*H_2_CH_3_ at C-1, 58.5 C-1, 115.5 C-3, 169.1 C=O at C-1, 158.6 C-2, 120.2-129.5 -C_arom_., C-4 carbon may be merged with -C_arom_, 129.9, 131.3, 132.7, 133.1, 133.6, 134.2, 141.0, 149.1 *ipso*-C’s.

*(2E)-ethyl-2-(2-(2,4-dinitrophenylhydrazono)-4-(naphthalen-2-yl)-6-(4-nitrophenyl) cyclohex-3-enecarboxylate, compound 23*

IR (KBr) ν (cm^-1^): 3324, 3088, 2922, 2858, 1735, 1640, 1518, 1324, 828, 742, 705, 630; ^1^H NMR (δ ppm), (J Hz): 0.91 (3H, t, CH_2_C*H*_3_ at C-1, J = 6.9), 3.14-3.19 (2H, H_5_, m), 3.67-3.73 (1H, H_6_, m), 3.94 (2H, q, C*H*_2_CH_3_ at C-1, J = 7.0), 4.17 (1H, H_1_, d, J = 13.2), 6.76 (1H, s, H_3_), 7.21-8.78 (14H, m, H_arom_.), 9.96 (1H, bs, NH); In the D_2_O exchanged ^1^H NMR spectrum, a broad singlet at 9.96 ppm due to labile NH proton disappeared; ^13^C NMR (δ ppm): 13.6 CH_2_*C*H_3_ at C-1, 35.0 C-5, 43.3 C-6, 60.3 *C*H_2_CH_3 _at C-1, 59.2 C-1, 115.7 C-3, 169.8 C=O at C-1, 158.3 C-2, 121.3-129.7 -C_arom_., C-4 carbon may be merged with -C_arom_, 129.4, 132.3, 133.6, 134.2, 137.7, 145.4, 149.2 *ipso*-C’s.

*(2E)-ethyl-2-(2-(2,4-dinitrophenylhydrazono)-6-(3-chlorophenyl)-4-(naphthalen-2-yl)-cyclohex-3-enecarboxylate, compound 24*

IR (KBr) ν (cm^-1^): 3324, 3098, 3060, 2972, 2924, 2852, 1734, 1657, 1512, 1330, 820, 787, 747, 698; ^1^H NMR (δ ppm), (J Hz): 0.94 (3H, t, CH_2_C*H*_3_ at C-1, J = 7.1), 3.19-3.21 (2H, H_5_, m), 3.45-3.74 (1H, H_6_, m), 3.93 (2H, q, C*H*_2_CH_3_ at C-1, J = 7.2), 4.13 (1H, H_1_, d, J = 14.1), 6.77 (1H, s, H_3_), 7.21-8.77 (14H, m, H_arom_.), 9.94 (1H, bs, NH); In the D_2_O exchanged ^1^H NMR spectrum, a broad singlet at 9.94 ppm due to labile NH proton disappeared; ^13^C NMR (δ ppm): 13.7 CH_2_*C*H_3_ at C-1, 34.7 C-5, 43.3 C-6, 60.2 *C*H_2_CH_3_ at C-1, 58.7 C-1, 115.4 C-3, 169.3 C=O at C-1, 158.3 C-2, 124.8-129.1 -C_arom_., C-4 carbon may be merged with -C_arom_, 129.2, 131.4, 132.6, 133.2, 133.7, 134.2, 140.2, 149.3 *ipso*-C’s.

*Microbiology*

*Materials*

All the clinically isolated bacterial strains namely *Salmonella typhii, Klebsiella pneumoniae, Escherichia coli, Pseudomonas aeruginosa, Staphylococcus aureus, β-Hemolytic streptococcus and Micrococcus luteus *and also fungal strains namely *Aspergillus flavus, Aspergillus Niger, Mucor, Rhizopus and Microsporumgypseum *are obtained from Faculty of Medicine, Annamalai University, Annamalai Nagar, 608 002, Tamil Nadu, India.

*In-vitro antibacterial and antifungal activity*

The minimum inhibitory concentration (MIC) of μg/mL values is carried out by two-fold serial dilution method (22). The respective test compounds 17-24 are dissolved in dimethyl sulfoxide (DMSO) to obtain 1 mg mL^-1^ stock solution. Seeded broth (broth containing microbial spores) is prepared in NB from 24 h old bacterial cultures on nutrient agar (Hi-media, Mumbai) at 37 ± 1 °C while fungal spores from 1 to 7 days old Sabouraud agar (Hi-media, Mumbai) slant cultures were suspended in SDB. The colony forming units (cfu) of the seeded broth are determined by plating technique and adjusted in the range of 10^4^-10^5^ cfu/mL. The final inoculums size was 10^5^cfu/mL for antibacterial assay and 1.1-1.5 X 10^2^ cfu/mL for antifungal assay. Testing is performed at pH of 7.4 ± 0.2 for bacteria (NB) and at a pH of 5.6 for fungi (SDB). Exactly 0.4 mL of the solution of test compound was added to 1.6 mL of seeded broth to form the first dilution. One mL of this was diluted with a further 1 mL of seeded broth to give the second dilution and so on till six of such dilutions are obtained. A set of assay tubes containing only seeded broth was kept as control. The tubes were incubated in BOD incubators at 37 ± 1°C for bacteria and 28 ± 1°C for fungi. The minimum inhibitory concentrations (MICs) are recorded through visual observations after 24 h (for bacteria) and 72-96 h (for fungi) of incubation. Ciprofloxacin is used as standard for bacterial studies and Fluconazole is used as standards for fungal studies.

## Results and Discussion

*Chemistry*

**Figure 2:**
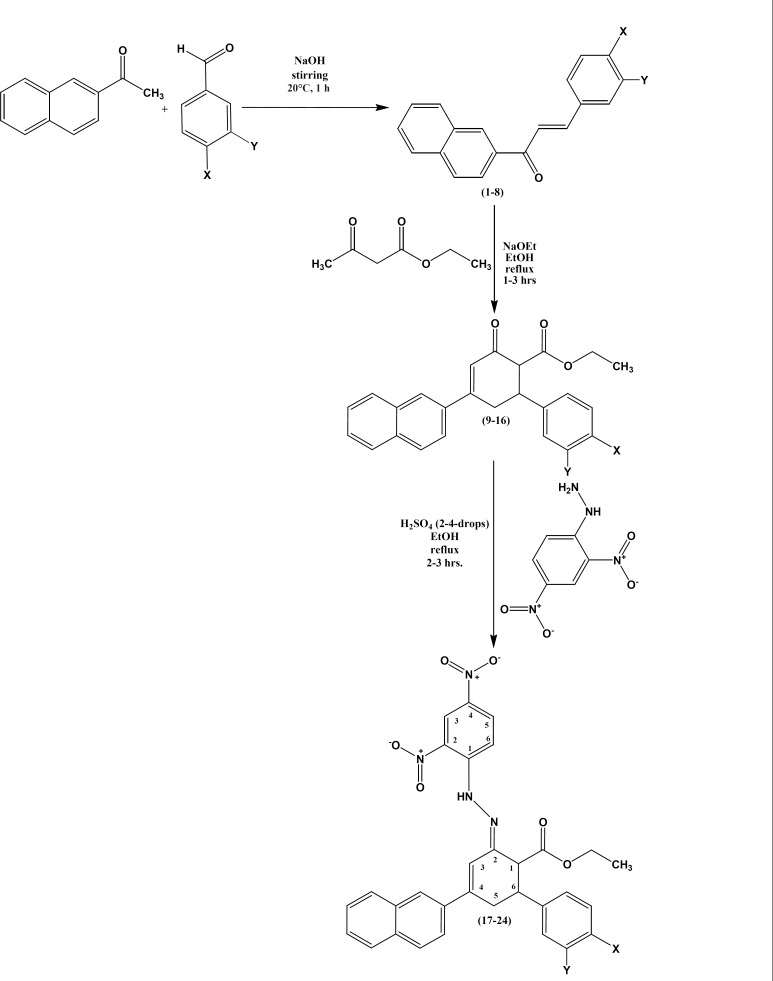
Synthesis of ethyl 4-(naphthalen-2-yl)-2-oxo-6-phenylcyclohex-3-enecarboxylates and (2*E*)-ethyl 2- (2-(2,4-dinitrophenyl)hydrazono)-4-(naphthalen-2yl)-6-arylcyclohex-3-enecarboxylates

Hydrazones, containing an azomethine -NHN=CH- proton, are synthesized by heating appropriate substituted hydrazines/hydrazides with aldehydes and ketones in solvents like ethanol, methanol, tetrahydrofuran, butanol, glacial acetic acid and ethanol-glacial acetic acid. Another synthetic route for the synthesis of hydrazones is the coupling of aryl diazonium salts with active hydrogen compounds. The reaction route for the synthesis of (2E)-ethyl-2-(2-(2, 4-dinitrophenyl)hydrazono)-4-(naphthalen-2-yl)-6-arylcyclohex-3-enecarboxylates 17-24 is as follows: (E)-1-(naphthalen-2-yl)-3-arylprop-2-ene-1-ones 1-8 are synthesized by the Claisen-Schmidt condensation of 2-acetyl naphthalene and substituted with benzaldehydes in the presence of alcoholic sodium hydroxide. Treatment of (E)-1-(naphthalen-2-yl)-3-arylprop-2-ene-1-ones 1-8 with ethyl acetoacetate in the presence of sodium ethoxide in refluxing ethanol (Figure 2 and Table 1) afford ethyl 4-(naphthalen-2-yl)-2-oxo-6-arylcyclohex-3-enecarboxylates 9-16. Compounds 9-16 on reaction with 2, 4-dinitrophenylhydrazine in the presence of 2-4 drops of sulfuric acid in refluxing ethanol yields the respective nitro hydrazone derivatives, (2E)-ethyl-2-(2-(2, 4-dinitrophenyl) hydrazono)-4-(naphthalene-2-yl)-6-arylcyclohex-3-enecarboxylates 17-24. The structures of all the synthesized ethyl 4-(naphthalen-2-yl)-2-oxo-6-arylcyclohex-3-enecarboxylates 9-16 are confirmed by m.p.’s, FT-IR, MS, 1H NMR and 13C NMR spectral studies and elemental analysis. Moreover, (2*E*)-ethyl-2-(2-(2,4-dinitrophenyl)hydrazono)-4-(naphthalen-2-yl)-6-arylcyclohex-3-ene carboxylates 17-24 are confirmed by m.p.’s, elemental analysis, FT-IR, MS, ^1^H NMR, D_2_O exchanged ^1^H NMR, ^13^C NMR, two dimensional ^1^H-^1^H HOMOCOR and ^1^H-^13^C HSQC spectral studies.

**Table 1:** Physical and analytical data of ethyl 4-(naphthalene-2-yl)-2-oxo-6-aryl cyclohex-3-enecarboxylates 9-16 and (2E)-ethyl-2-(2-(2,4-dinitrophenyl)hydrazono)-4-(naphthalen-2-yl)-6-arylcyclohex-3-enecarboxylates (compounds 9-24).

**Entry**	**X**	**Y**	**Reaction time (h)**	**Yield (%)**	**m.p. (°C)**	**Elemental analysis**			**m/z (M+1)** ^+•^ **Molecular formula**
						**C Found (Calculated)**	**H Found (Calculated)**	**N Found (Calculated)**	
**9**	H	H	2	80	146	81.01 (81.06)	5.92 (5.99)	-	371 [C_25_H_22_O_3_]
**10**	CH_3_	H	3	78	110	81.17 (81.22)	6.25 (6.29)	-	385 [C_26_H_24_O_3_]
**11**	F	H	1	82	126	77.24 (77.30)	5.40 (5.45)	-	389 [C_25_H_21_FO_3_]
**12**	OCH_3_	H	3	80	122	77.91 (77.98)	6.00 (6.04)	-	401 [C_26_H_24_O_4_]
**13**	Cl	H	1	75	114	74.12 (74.16)	5.20 (5.23)	-	405 [C_25_H_21_ClO_3_]
**14**	Br	H	1	78	126	66.76 (66.82)	4.67 (4.71)	-	449 [C_25_H_21_BrO_3_]
**15**	NO_2_	H	2	65	132	72.22 (72.28)	5.07 (5.10)	3.33 (3.37)	416 [C_25_H_21_NO_5_]
**16**	H	Cl	2	78	98	74.10 (74.16)	5.15 (5.23)	-	405 [C_25_H_21_ClO_3_]
**17**	H	H	2	82	125	67.57 (67.63)	4.72 (4.76)	10.12 (10.18)	551 [C_31_H_26_N_4_O_6_]
**18**	CH_3_	H	2	85	132	68.03 (68.07)	4.94 (5.00)	9.86 (9.92)	565 [C_32_H_28_N_4_O_6_]
**19**	F	H	3	75	117	65.42 (65.49)	4.37 (4.43)	9.78 (9.85)	569 [C_31_H_25_FN_4_O_6_]
**20**	OCH_3_	H	2	80	110	66.16 (66.20)	4.79 (4.86)	9.59 (9.65)	581 [C_32_H_28_N_4_O_7_]
**21**	Cl	H	3	72	128	63.59 (63.65)	4.29 (4.31)	9.52 (9.58)	586 [C_31_H_25_ClN_4_O_6_]
**22**	Br	H	3	78	176	59.08 (59.15)	3.94 (4.00)	8.88 (8.90)	629 [C_31_H_25_BrN_4_O_6_]
**23**	NO_2_	H	3	70	164	62.47 (62.52)	4.19 (4.23)	11.72 (11.76)	596 [C_31_H_25_N_5_O_8_]
**24**	H	Cl	3	78	121	63.60 (63.65)	4.26 (4.31)	9.50 (9.58)	586 [C_31_H_25_ClN_4_O_6_]

**Figure 3:**
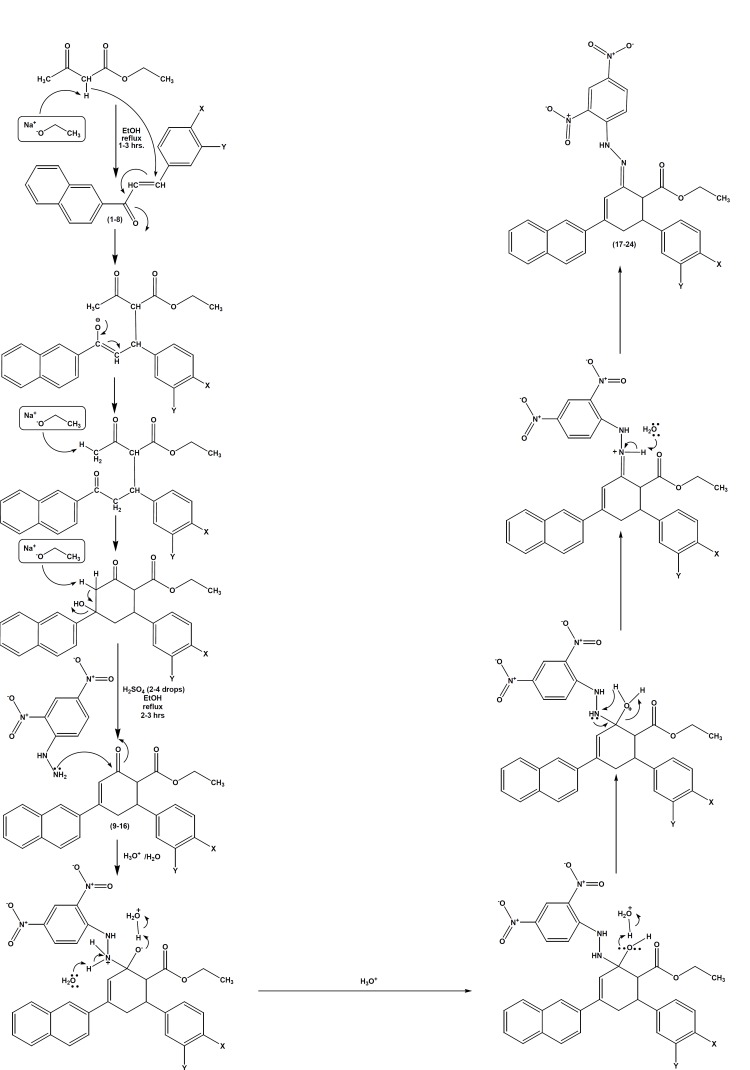
Mechanistic pathway for the formation of ethyl 4-(naphthalen-2-yl)-2-oxo-6-phenylcyclohex-3-enecarboxylates and (2*E*)-ethyl2- (2-(2,4-dinitrophrnyl)hydrazono)-4-(naphthalen-2-yl)-6-arylcyclohex-3-enecarboxylates

The reaction mechanism (Figure 3) involves the formation of Michael addition product by ethyl acetoacetate with (E)-1-(naphthalen-2- yl)-3-arylprop-2-ene-1-ones 1-8 in the presence of base, sodium ethoxide. Later, the addition product undergoes intramolecularaldol reaction in the presence of sodium ethoxide base, to give ethyl 4-(naphthalen-2-yl)-2-oxo-6-arylcyclohex-3-enecarboxylates 9-16. Ethyl 4-(naphthalen-2-yl)-2-oxo-6-arylcyclohex-3-enecarboxylates 9-16 contain *β*-dicarbonyl system on reaction with 2,4-dinitrophenylhydrazine in the presence of 2-4 drops of sulfuric acid in refluxing ethanol yields (2E)-ethyl-2-(2-(2,4-dinitrophenyl)hydrazono)-4-(naphthalen-2-yl)-6-arylcyclohex-3-enecarboxylates 17-24.

 This reaction can be described as a condensation reaction, with two molecules join together with loss of water. It is also called addition-elimination reaction: nucleophilic addition of the –NH_2 _group to the C=O carbonyl group, followed by the removal of a water molecule. In the present reaction, 2, 4-dinitrophenylhydrazine does not react with other carbonyl-containing functional groups such as ester. For ester, there is resonance associated stability as a lone pair of electrons interacts with the p-orbital of the carbonyl carbon resulting in increased delocalization in the molecule. This stability would be lost by addition of a reagent to the carbonyl group. Hence, these compounds are more resistant to addition.

Ethyl 4-(naphthalen-2-yl)-2-oxo-6-phenyl cyclohex-3-enecarboxylate 9 is chosen as the representative compound to discuss the spectral data of Ethyl 4-(naphthalen-2-yl)-2-oxo-6-arylcyclohex-3-enecarboxylates 9-16. FT-IR spectrum of Ethyl 4-(naphthalen-2-yl)-2-oxo-6-phenylcyclohex-3-enecarboxylate 9 shows two strong characteristic absorptions at 1738 and 1659 cm^-1^due to ester ketone and carbonyl functional groups respectively. The band at 1606 cm^-1^ is due to the presence of C=C stretching frequency. The absorption frequency at 3057 cm^-1^ is assigned to aromatic C-H stretching vibration and the absorption frequencies at 2987 and 2929 cm^-1^ is assigned to aliphatic C-H stretching vibration. The observed ester carbonyl, ketone and C=C stretching vibrational bands, are supporting evidence for the formation of synthesized compound 9. In the ^1^H NMR spectrum of 9, a triplet was observed at 0.92 ppm (J = 7.1 Hz) corresponding to three protons and this signal is due to the ester methyl protons at C-1. A quartet observed at 3.91 ppm (J = 7.0 Hz) corresponding to two protons and this signal is due to the ester methylene protons at C-1. Two multiplets are obtained in the range of 3.22-3.28 and 3.65-3.73 and they are due to the H-5 and H-6 protons. The doublet at 4.17ppm (J = 13.4 Hz) has been assigned to H-1 proton. The singlet observed in downfield region at 6.73 ppm is due to H-3 proton. The aromatic protons appeared as a multiplet in the range of 7.24-8.33ppm. Two ^13^C resonances at 194.2 and 169.2 ppm are assigned to C-2 carbonyl carbon and ester carbonyl carbon respectively. The^ 13^C resonances at 35.1 and 43.8 ppm are due to the C-5 and C-6 carbons respectively. The ^13^C resonance observed at 58.7 and 13.7 ppm are assigned to ester methylene and methyl carbons at C-1 respectively. The signal observed at 59.9 ppm is assigned to C-1 carbon, whereas the signal at 123.1 ppm is assigned to C-3 carbon. The aromatic carbons are observed in the range of 123.4-128.8 ppm. C-4 carbon may be merged with the aromatic carbons. The remaining ^13^C signals at 132.7, 133.6, 134.3 and 141.5 ppm are due to *ipso *carbons.

In order to discuss the spectral data of (2E)-ethyl-2-(2-(2,4-dinitrophenyl)hydrazono)-4-(naphthalen-2-yl)-6-arylcyclohex-3-enecarboxylates 17-24, (2*E*)-ethyl-2-(2-(2,4-dinitrophenylhydrazono)-4-(naphthalene-2-yl)-6-phenylcyclohex-3-enecarboxylate 17 is chosen as the representative compound.

FT-IR spectrum of (2E)-ethyl-2-(2-(2,4-dinitrophenylhydrazono)-4-(naphthalen-2-yl)-6-phenylcyclohex-3-enecarboxylate 17 shows characteristic absorption frequency at 3323 cm^-1^ which suggests the presence of -NH functional group. The absorption frequency at 1656 cm^-1^ is assigned to C=N stretching vibration. The band at 1512 cm^-1^ is due to the presence of C=C stretching frequency. Nitro functional groups show characteristic stretching frequency around 1330 cm^-1^. Besides these, aromatic CH stretching frequencies are observed at 3099 and 3060 cm^-1^ and the aliphatic CH stretching frequencies are observed at 2989, 2924 and 2847 cm^-1^. The observed -NH, C=N, C=C and NO_2_ stretching vibrational bands are supporting evidence for the formation of synthesized compound 17. In the ^1^H NMR spectrum of 17, a triplet was observed at 0.92 ppm (J = 7.0 Hz) corresponding to three protons and this signal is due to ester methyl protons at C-1. A quartet observed at 3.91 ppm (J = 7.0 Hz) corresponding to two protons and this signal is due to ester methylene protons at C-1. Two multiplets are obtained in the range 3.20-3.21 and 3.66-3.72 and they are due to H-5 and H-6 protons. The doublet at 4.16 ppm (J = 13.4 Hz) has been assigned to H-1 proton. The singlet observed in downfield region at 6.73 ppm is due to H-3 proton. The aromatic protons appeared as a multiplet in the range of 7.25-8.80 ppm. The aromatic protons due to dinitrophenyl part are observed in the range of 8.12-8.80 ppm. H-3 proton of 2,4-dinitrophenyl part gives signal as a doublet at 8.80 ppm (J = 2.5 Hz). H-5 proton of 2,4-dinitrophenyl part gives signal as a multiplet around 8.32-8.23 ppm. H-6 proton of 2,4-dinitrophenyl part gives signal as a doublet at 8.12 ppm (J = 7.3Hz). The labile NH proton (exchangeable with D_2_O) appears as a broad singlet at 9.97 ppm. In the HOMOCOSY spectrum of 17, the signal at 0.92 ppm shows cross peak with the signal at 3.91 ppm and vice versa. From the above cross peaks, it is confirmed that the triplet observed at 0.92 ppm corresponds to ester methyl protons at C-1, whereas the quartet at 3.91 ppm corresponds to ester methylene protons at C-1. The signal at 4.16 ppm gives cross peak with multiplet at 3.66-3.72. Similarly, the multiplet at 3.66-3.72 gives cross peaks with the signal at 4.16 ppm as well as the signal at 3.20-3.21 ppm. Furthermore, the signal at 6.73 ppm gives correlations with the signal at 3.20-3.21 ppm and vice versa. From the above correlations, it reveals that the singlet at 6.73 ppm corresponds to H-3 proton and the multiplet at 3.66-3.72 ppm corresponds to H-6 proton respectively. The doublet at 4.16 ppm is conveniently assigned to H-1 proton and the multiplet at 3.20-3.21 ppm is unambiguously assigned to H-5 proton.

In the ^13^C NMR spectrum of 17, five resonances in the aliphatic region at 13.8, 35.1, 43.8, 58.7 and 59.9 ppm are observed. They are all due to CH_2_*C*H_3_ at C-1, C-5, C-6, C-1 and *C*H_2_CH_3_ at C-1 respectively. C-3 carbon resonates at 115.5 ppm. The remaining ^13^C resonances in quaternary carbon signals at 169.2 and 158.7 ppm are due to C=O at C-1 and C-2 carbon respectively.

The aromatic carbons are observed in the range of 123.1-128.8 ppm. The carbon signals due to aromatic carbons of 2,4-dinitrophenyl part are merged with the aromatic carbons of naphthyl and phenyl rings at the position of 4 and 6 in cyclohexene ring. C-4 carbon may be merged with aromatic carbon signals. The ^13^C resonances at 129.5, 132.7, 133.6, 134.3, 141.5 and 149.5 ppm are due to *ipso *carbons. In the HSQC spectrum of 17, one bond correlation (13.8/0.92 ppm) is observed between CH_2_*C*H_3_ at C-1 and CH_2_C*H*_3_ at C-1. The ^13^C resonance at 59.9 has correlations with ester methylene protons, C*H*_2_CH_3_ at C-1. Another one-bond correlation (35.1/3.20-3.21 ppm) is observed between C-5 and H-5. The ^13^C resonance at 43.8 ppm has a one-bond correlation with a multiplet around 3.66-3.72 ppm. Hence, the signal at 43.8 ppm corresponds to C-6 carbon whereas the multiplet at 3.66-3.72 ppm is assigned to H-6 proton. The other aliphatic carbon which resonances at 58.7 ppm, shows a one-bond correlation with a doublet at 4.16 ppm. From this correlation, it is revealed that the doublet at 4.16 ppm corresponds to H-1 proton of the cyclohexenone moiety and the ^13^C signal at 58.7 is assigned to C-1 carbon. The ^13^C resonance at 115.5 ppm has correlations with singlet at 6.73 ppm. So the signal at 6.73 ppm is conveniently assigned to H-3 proton and the carbon signal at 115.5 ppm is assigned to C-3. In the HSQC, the ^13^C resonances at 169.2 and 158.7 ppm has no correlations with protons and hence it is due to quaternary carbons, ester C=O at C-1 and C=N respectively. The C-4 carbon may be merged with the aromatic carbons. Among the quaternary carbons, the 13C resonances at 129.5, 132.7, 133.6, 134.3, 141.5 and 149.5 are due to *ipso *carbons.

*Antibacterial activity*

Novel (2E)-ethyl-2-(2-(2,4-dinitrophenyl)hydrazono)-4-(naphthalen-2-yl)-6-arylcyclohex-3-enecarboxylates 17-24 are tested for their antibacterial activity *in*-*vitro *against *S. typhi, K.pneumoniae, E.coli, P.aeruginosa, S.aureus, β-H.streptococcus and M.luteus. *Ciprofloxacin is used as standard bactericidal drug. Minimum inhibitory concentration (MIC) in μg/mL values is reproduced in Table 2. 

**Table 2:** *In-vitro *antibacterial activity (MIC) values for (2E)-ethyl-2-(2-(2,4-dinitrophenyl)hydrazono)-4-(naphthalen-2-yl)-6-arylcyclohex-3-enecarboxylates (compounds 17-24)

**Compounds**	**Minimum Inhibitory Concentration (MIC) in μg/mL**						
	***S.typhii***	***K. pneumoniae***	***E.coli***	***P. aeruginosa***	***S.aureus***	***β-H. streptococcus***	***M.luteus***
**17**	100	100	200	100	50	50	6.25
**18**	200	100	-	100	12.5	6.25	12.5
**19**	6.25	6.25	25	6.25	12.5	6.25	25
**20**	200	-	200	200	6.25	12.5	12.5
**21**	12.5	25	6.25	12.5	12.5	25	6.25
**22**	25	25	100	6.25	100	25	50
**23**	25	12.5	50	6.25	6.25	25	12.5
**24**	25	12.5	25	25	12.5	6.25	50
Ciprofloxacin	25	25	50	25	25	50	25

^a^ - No inhibition even at higher concentration *i.e., *at 200 μg/mL

A close survey of the MIC values indicates that all the compounds 17-24 exhibited a varied range (6.25-200 μg/mL) of antibacterial activity against all the tested bacterial strains except compounds 18 and 20 against *E.coli *and *K.pneumoniae *respectively which do not have activity even at a maximum concentration of 200 μg/mL. Compounds 18 and 20, which have electron donating methyl/methoxy substituent at the *para *position of phenyl rings attached to C-6 of cyclohexenone moiety, showed moderate activity against Gram-negative bacteria and showed good activity against Gram-positive bacteria at a MIC value in the range of 6.25-12.5 μg/mL against *S. aureus*, *β.H.streptococcus *and *M. luteus*. Compound 17 which has no substitution at the phenyl ring attached to C-6 of cyclohexenone moiety, exhibited moderate activity against all the tested bacterial strains. Compound 19 shows excellent antibacterial activity against *S. typhi*, *K.pneumoniae, P.aeruginosa *and *β.H.streptococcus *at a MIC value of 6.25 μg/mL which is a four-fold increase in activity with that of Ciprofloxacin. Similarly, compound 21 which has chloro substitution at the *para *position of phenyl rings attached to the C-6 of cyclohexenone moiety, exerted excellent activity against *E.coli *and *M. luteus *at a MIC value of 6.25 μg/mL and exerted a MIC value of 12.5 μg/mL against *S. typhi*, *P.aeruginosa *and *S. aureus *respectively. In addition, compound 24, a *meta*chloro substituted compound exhibited good antibacterial activity against *K. pneumonia *and *S. aureus *at a MIC value of 12.5 μg/mL whereas it showed activity at a MIC of 6.25 μg/mL against *β.H.streptococcus. *Bulky bromo substitution at the *para *position of phenyl rings attached to C-6 of cyclohexenone moiety in compound 22 is active against *P. aeruginosa*. Moreover, compound 23 which has nitro substitution at the *para*position of phenyl rings attached to C-6 of cyclohexenone moiety exerted excellent activity against *P. aeruginosa *and *S. aureus *at a MIC value of 6.25 μg/mL and it showed good activity against *K. pneumonia *at a MIC value of 12.5 μg/mL. All the electron withdrawing substituent namely fluoro, chloro, bromo or nitro compounds such as 19, 21-24 exerted strong antibacterial activity against all the tested strains when compared to standard drug Ciprofloxacin whereas electron donating substituent namely methyl or methoxy compounds such as 18 and 20 show good activity against Gram-positive bacteria at a MIC value in the range of 6.25-12.5 μg/mL.

*Antifungal activity*

The *in*-*vitro *antifungal activity of (2E)-ethyl-2-(2-(2,4-dinitrophenyl)hydrazono)-4-(naphthalen-2-yl)-6-arylcyclohex-3-enecarboxylates 17-24 was studied against the fungal strains *viz., A. flavus, A. Niger, Mucor, Rhizopus and Microsporumgypseum. *Fluconazole was used as a standard drug. Minimum inhibitory concentration (MIC) in μg/mL values is reproduced in Table 3.

**Table 3:** *In-vitro *antifungal activity (MIC) values for (2E)-ethyl-2-(2-(2,4-dinitrophenyl) hydrazono)-4-(naphthalen-2-yl)-6-arylcyclohex-3-enecarboxylates (compounds 17-24).

**Compounds**	**Minimum Inhibitory Concentration (MIC) in μg/mL**				
	***A. flavus***	***A. Niger***	***Mucor***	***Rhizopus***	***M. gypseum***
**17**	200	100	100	200	-^a^
**18**	25	25	6.25	12.5	25
**19**	6.25	6.25	25	12.5	6.25
**20**	25	12.5	12.5	6.25	25
**21**	12.5	6.25	25	-^a^	6.25
**22**	6.25	6.25	25	12.5	25
**23**	-a	50	6.25	100	25
**24**	6.25	6.25	12.5	100	25
Fluconazole	50	50	25	25	25

^a^ - No inhibition even at higher concentration *i.e*., at 200 μg/mL

 A close survey of the MIC values indicates that all the compounds 17-24 exhibited a varied range (6.25-200 μg/mL) of antifungal activity against all the tested fungal strains except compounds 17, 21 and 23 which do not have antifungal activity against *M. gypseum, Rhizopus*and *A. flavus*respectively even at a high concentration of 200 μg/mL. Compound 17, having no substitution at the phenyl rings attached to C-6 carbon of cyclohexenone moiety exerted moderate activity against all the tested fungal strains, whereas methyl substituted compound 18 exerted good antifungal activities against *Mucor, Rhizopus*at a MIC value of 6.25 and 12.5 μg/mL respectively. Similar results are noticed for electron donating methoxy substituent compound 20 and exerted antifungal activity against *Rhizopus*at a MIC of 6.25 μg/mL, whereas it showed potent activity against *A. Niger *and *Mucor*at a MIC of 12.5 μg/mL. Compound 19 showed excellent antifungal activity against all the tested fungal strains and it showed MIC value of 6.25-25 μg/mL. Similarly, chloro substituted compound 21 and nitro substituted compound 23 exhibited good antifungal activities against all the tested strains except against *Rhizopus*and *A .flavus*respectively which did not have potent activity even at a higher concentration of 200 μg/mL. Compound 22, which has bulky bromo substitution at the *para*position of the phenyl rings attached to C-6 carbon of cyclohexenone moiety exerted good antifungal activity against all the tested fungal strains. All the naphthylhydrazone derivatives 17-24 were more potent and exerted good antifungal activity when compared to the standard drug Fluconazole. Compounds with electron withdrawing fluoro, chloro, bromo or nitro substituent at the phenyl rings or compounds that having electron donating methyl or methoxysubstitutents attached to C-6 of cyclohexenone moiety, exerted excellent activity against all the tested fungal strains.

## Conclusion

In crisp, an array of novel of (2E)-ethyl-2-(2-(2,4-dinitrophenyl)hydrazono)-4-(naphthalen-2-yl)-6-arylcyclohex-3-enecarboxylates 17-24 are synthesized and characterized by their physical and analytical data. This reaction may have wide applicability in building a variety of heterocycles by choosing 4-(naphthalen-2-yl)-2-oxo-6-arylcyclohex-3-enecarboxylates as synthon, which has three versatile functional groups *i.e*., ketone, olefin and ester for the synthesis of structurally diverse organic compounds. The microbiological screening studies carried out to evaluate the antibacterial and antifungal potencies of the newly synthesized (2E)-ethyl-2-(2-(2,4-dinitrophenyl)hydrazono)-4-(naphthalen-2-yl)-6-arylcyclohex-3-enecarboxylates 17-24 are clearly known from Tables 2 and 3. A close inspection of the *in-vitro *antibacterial and antifungal activity profile in differently electron donating (CH_3_ and OCH_3_) and electron withdrawing (-F, -Cl, Br and –NO_2_) functional group substituted phenyl rings of novel (2E)-ethyl-2-(2-(2,4-dinitrophenyl)hydrazono)-4-(naphthalen-2-yl)-6-arylcyclohex-3-enecarboxylates 17-24 exerted strong anti-bacterial activity against all the tested bacterial strains. Compound 17 against *M. luteus, *compound 18 against *β.H.streptococcus*, compound 19 against *S. typhi, K.pneumoniae, P. aeruginosa, β.H.streptococcus*and *M. luteus*, compound 20 against *S. aureus, *compound 21 against *K. pneumoniae*and *M. luteus*, compound 22 against *P. aeruginosa*, compound 23 against *P. aeruginosa*and *S. aureus*and compound 24 against *β.H.streptococcus*exerted excellent antibacterial activity at a MIC value of 6.25 μg/mL. Results of the anti-fungal activity study show that the nature of substituents on the phenyl ring *viz*., methyl, fluoro, methoxy, chloro, bromo and nitro functions at the *para*positions of the aryl moieties are determinant for the nature and extent of the anti-fungal activity of all the synthesized compounds 17-24 over fungal strains namely *A. flavus, A. Niger, Mucor, Rhizopus*and *M. gypseum. *Compound 18 against *Mucor*, compound 19 against *A. flavus, A. Niger, M. gypseum, *compound 20 against *Rhizopus, *compound 21 against *A. Niger, M. gypseum, *compound 22 and 24 against both *A. flavus, A .Niger *and compound 23 against *Mucor*exhibited excellent antifungal activity a MIC value of 6.25 μg/mL. These antifungal results are about eight to four-fold increase in activity exerted by naphthylhydrazone derivatives in comparison with their standard drug, fluconazole. Results of the antimicrobial activity show that electron withdrawing substitutents like fluoro, chloro, bromo and nitro substituted derivatives exerted excellent antibacterial and antifungal activities, since electron withdrawing substituent increases the lipophilicity due to the strong electron withdrawing capability (23). Moreover, electron withdrawing substitutents namely fluorine substitution was commonly used in contemporary medicinal chemistry to improve metabolic stability, bioavailability and protein ligand interactions (24). These observations may promote a further development of our research in this field. Furthermore, the observed marked antibacterial and antifungal activities of this group of naphthylhydrazone derivatives may be considered as the key steps for the building of novel chemical entities with comparable pharmacological profiles to that of the standard drugs.
